# Optical Detection of Fat Concentration in Milk Using MXene-Based Surface Plasmon Resonance Structure

**DOI:** 10.3390/bios12070535

**Published:** 2022-07-18

**Authors:** Abdulkarem H. M. Almawgani, Malek G. Daher, Sofyan A. Taya, Mohammad Mashagbeh, Ilhami Colak

**Affiliations:** 1 Electrical Engineering Department, College of Engineering, Najran University, Najran 66462, Saudi Arabia; ahalmawgani@nu.edu.sa; 2 Physics Department, Islamic University of Gaza, P.O. Box 108, Gaza P860, Palestine; malekjbreel20132017@gmail.com; 3 Department of Mechatronic Engineering, University of Jordan, Amman 11942, Jordan; m.mashagbeh@ju.edu.jo; 4 Department of Electrical and Electronics Engineering, Nisantasi University, Istanbul 34398, Turkey; ilhcol@gmail.com

**Keywords:** surface plasmon resonance, fat concentration in milk, MXene, sensitivity

## Abstract

MXene (Ti_3_C_2_T_x_) has emerged very recently as an interacting material for surface plasmon resonance (SPR) configuration. It was discovered that Ti_3_C_2_T_x_ can facilitate the adsorption of biomolecules due to its higher binding energies, stronger interaction between matter and light, and larger surface area. In this work, a two-dimensional Ti_3_C_2_T_x_ and silicon layer-based SPR refractometric sensor is proposed for the sensitive and fast detection of milk fat concentration due to the high significance of this issue to people all over the world. The proposed SPR structure employs BK7 (BK7 is a designation for the most common Borosilicate Crown glass used for a variety of applications in the visible range) as a coupling prism and silver as a metal layer. The layer thicknesses and the number of Ti_3_C_2_T_x_ sheets are optimized for the highest performance. The highest reached sensitivity is 350 deg./RIU with 50 nm silver and 4 nm silicon with a monolayer of Ti_3_C_2_T_x_, which is ultra-high sensitivity compared to the latest work that utilizes SPR configuration. The proposed SPR-based sensor’s ultra-high sensitivity makes it more attractive for usage in a variety of biosensing applications.

## 1. Introduction

The surface plasmon resonance (SPR) sensor is a plasmonic device utilized in biosensing and chemical applications, such as food safety, drug diagnostics, and medical diagnostics [1,2,3,4,5]. SPR sensors, among other modern sensing techniques, are the perfect technology for sensing applications because of their worthwhile properties, such as the ability to perform real-time sensing in a label-free platform, their quick response, and high sensitivity. The metals that can support plasmons in SPR sensors are silver, gold, indium, aluminum, copper, and sodium. Because of its higher sensitivity, durability, biocompatibility and superior stability, biocompatibility, and higher sensitivity, gold is preferred over silver in SPR sensors [6,7,8]. Silver, on the other hand, can be used to minimize oxidation by coating another layer above it [6,7,8]. The principle of an SPR sensor is to determine the variation in the RI of an analyte when biomolecules interact on the sensor surface. The phase matching of the evanescent wave created by the transverse magnetic (TM) light and surface plasmon (SP) wave determines the SPR condition. When this condition is achieved, a dip is noticed in the reflectance profile. The angular position of the reflectance dip depends on many parameters, including the used prism, incident light wavelength, 2D materials, metal, and binding of the biomolecule [9,10,11,12]. The reflectance curve is employed to determine the sensing performance of the SPR sensor.

Due to their unique electrical and optical properties, 2D nanomaterials like black phosphorus (BP), graphene (G), and transition-metal dichalcogenides (TMDGs) have sparked a lot of interest in SPR sensors [13]. The employment of BP, G, and TMDGs in SPR sensors has attracted much interest in 2D nanomaterials. SPR is an attractive procedure for detecting biomolecules because it uses 2D nanomaterials that interact well with the biomolecules of the sensing material [14]. The number of 2D nanomaterial layers can be varied to realize desired electronic properties for enhanced biosensing. These 2D nanomaterials can be manufactured using a chemical vapor deposition approach (CVD) [15]. The CVD technique is costly and inefficient. The fabrication of 2D nanomaterials is a key issue that has to be solved before it is extensively used. A recent paper reported a new method based on electrospinning to fabricate 2D inorganic materials. Such a method has many advantages, such as continuous production, low production cost, and safe production [16]. An SPR sensor is constructed with many layers of 2D nanomaterials on the metal layer. The scientific community has recently become interested in constructing heterostructures containing 2D nanomaterial on a single chip in order to improve their performance [9]. The electrical and optical properties of 2D heterostructures differ from those of their constituent material, which is advantageous for SPR applications [17]. The issue of aligning and precisely stacking different 2D materials when creating such heterostructures increases the complexity of the SPR device and cost. However, adding a nanolayer of silicon (Si) over the metal layer in an SPR sensing device to increase its sensitivity is a proven method. Many researchers have employed Si in the last ten years to reduce the complexity and cost of SPR sensors that use 2D nanomaterials for biosensing applications [18]. It is a dielectric substance with a high refractive index (RI) that enhances the intensity of the TM field at the sensing interface [19]. SPR structures with Si and graphene have been proposed by Verma et al. [19]. They confirmed that employing graphene layers in SPR-based biosensors can enhance biomolecule adsorption. They also confirmed that the use of Si thin film between the metal and graphene layers significantly improves the SPR structure performance. In a recent work, the authors proposed the use of Si and TMDGs on a metal layer in an SPR sensor and the sensitivity attained a considerable enhancement [20]. A BlueP/MoS_2_-based SPR biosensor was investigated by A Srivastava et al. [21]. They concluded that the BlueP/MoS_2_-based SPR biosensor had a high sensitivity and could detect minor changes in the RI of an analyte in comparison to a traditional SPR sensor. BlueP/MoS_2_ was employed as an interaction layer for analyte attachment, which improved sensitivity even more. The highest sensitivity was 2.4 times higher than that of a standard SPR sensor.

A new 2D nanomaterial, Ti_3_C_2_T_x_, has emerged very recently as an interacting layer in SPR configuration [22]. Researchers have discovered that Ti_3_C_2_T_x_ is a promising 2D material for biomolecule sensing [22,23]. This is because of its unique properties, such as higher binding, strong carrier confinement, smaller work function, layered nature, mechanical and chemical stability, and larger surface area [23]. Ti_3_C_2_T_x_ has a plasmonic property that can be altered by changing the surface terminations [24]. However, with careful control of its surface termination, Ti_3_C_2_T_x_ can be used in biomolecule SPR sensors. An SPR biosensor utilizing few layers of Ti_3_C_2_T_x_ and different metals was proposed by L. Wu et al. [25]. The highest sensitivities were found for Ag, Au, Cu, and Al structures when the Ti_3_C_2_T_x_ nanomaterials had 7, 4, 9, and 12 layers, respectively. Ti_3_C_2_T_x_, WS_2_, and black phosphorus were theoretically presented in an SPR biosensor structure by Srivastava et al. [26] with a monolayer of each nanomaterial and a sensitivity of 190.22 deg./RIU was achieved. S. Pal et al. [27] recently presented an SPR biosensor based on a heterostructure of 2D BlueP/MoS_2_ and Ti_3_C_2_T_x_ for sensitivity improvement. For the proposed design using a CaF_2_ prism, the researchers achieved a maximum sensitivity of 203 deg./RIU. The advantage of the structure described in this paper over other sensors is the use of a very thin layer of dielectric material between the metal and Ti_3_C_2_T_x_ layers.

Milk and dairy-related products are the most prevalent components of several food products, so monitoring their quality is critical. Additives in milk, such as whey, added water, starch, formalin, formaldehyde and sucrose, sodium citrate, indigenous vegetable oils, and others have been detected and quantified by many researchers [28,29]. The concentrations of milk’s ingredients determine its RI. The RI of milk, for example, fluctuates when the fat concentration changes. Knowing the RI of milk can help to figure out how much fat is in it [30,31].

In this paper, we propose an SPR-based refractometric sensor that uses a thin layer of Si and Ti_3_C_2_T_x_ layered nanomaterial. The SPR refractometric sensor has a thin layer of Si on the top of a silver layer and a two-dimensional Ti_3_C_2_T_x_ layer is placed between the silver thin film and sensing medium to achieve higher sensitivity. The current study has many advantages, such as using a 2D nanomaterial (Ti_3_C_2_T_x_) with a dielectric material (Si) in an SPR structure and the subsequent high sensitivity. Moreover, to our knowledge, this is the first study of an SPR-based sensor for the detection of the fat concentration in milk.

## 2. Structure Consideration

**Figure 1:**
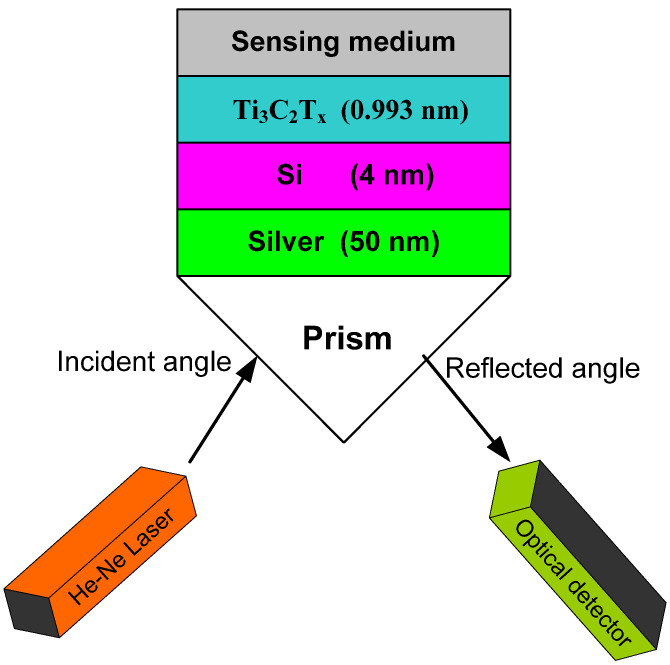
An SPR refractometric sensor consists of prism, silver, Si, and Ti_3_C_2_T_x_ layers.

The proposed structure has five layers: prism, Ag, Si, Ti_3_C_2_T_x_, and analyte medium as illustrated in Figure 1. The BK7 glass prism (index *n_p_*) is employed in this work. In a recent study, we demonstrated four different commonly used prisms, which are N-FK51A, 2S2G, SF10, and BK7. We found that structures with N-FK51A and BK7 had the highest sensitivity [32]. It was shown in the same study that sensor sensitivity can be enhanced when using a low refractive index prism. A Helium–Neon laser (λ = 632.8 nm) is assumed. For the detection of the fat concentration in milk, various concentrations have to be employed as analyte media. The layers of Ag, Si, and Ti_3_C_2_T_x_ have thicknesses of d_Ag_, d_S_, and d_M_ and the indices *n_Ag_*_,_ *n_S_*, and *n_M_*, respectively.

The RI of the BK7 glass prism depends on the light wavelength according to [33]

(1) $$n_{p}\left(\lambda \right)=\sqrt{1+\frac{d_{1}\;\lambda^{2}}{\lambda^{2}-e_{1}}+\frac{d_{2}\;\lambda^{2}}{\lambda^{2}-e_{2}}+\frac{d_{3}\;\lambda^{2}}{\lambda^{2}-e_{3}}}$$

where the values of the coefficients *d_i_* and *e_i_* are given in Table 1.

λ is inserted into Equation (1) in μm.

The Drude–Lorentz model is commonly employed to express the RI of metals as a function of λ. The RI of the silver layer is calculated as [34]

(2) $$n_{Ag}\left(\lambda \right)={\left(1-\frac{\lambda_{c}\;\lambda^{2}}{\lambda_{p}{}^{2}\left(\lambda_{c}+i\lambda \;\right)}\right)}^{1/2}$$

where $\lambda_{c}=1.761\times 10^{-5}\;m\;$ and $\lambda_{p}=1.454\times 10^{-7}\;m$ are the collision and plasma wavelengths of silver. The graphene layer RI is given by [35]

(3) $$n_{G}\left(\lambda \right)=3+\lambda \frac{C}{3}i$$

where *C* = 5.446 in 1/μm unit.

The RI of the Si layer is given by [36]

(4) $$n_{S}\left(\lambda \right)={\left(1+\frac{10.668493\;\lambda^{2}}{\lambda^{2}-{\left(0.301516485\right)}^{2}}+\frac{0.00304347\;\lambda^{2}}{\lambda^{2}-{\left(1.13475115\right)}^{2}}+\frac{1.54133408\;\lambda^{2}}{\lambda^{2}-{\left(1104.0\right)}^{2}}\right)}^{1/2}$$

SPR is a phenomenon that is limited to p-polarized light in natural materials with positive permittivity and permeability. Angular modulation was employed to study the proposed SPR device. The reflectivity of p-polarized incident waves was investigated using the multilayer reflection theory of Fresnel and the transfer matrix (TM) approach. The propagation constants of p-polarized incident light and surface plasmon must match for surface plasmon to be excited. Mathematically,

(5) $$\frac{\omega }{c}\sqrt{\varepsilon_{p}}\;sin\theta_{in}=\frac{\omega }{c}\sqrt{\frac{\varepsilon_{Ag}\varepsilon_{d}}{\varepsilon_{Ag}+\varepsilon_{d}}}$$

where *ε_p_*, *ε_Ag_*, and *ε_d_* are the relative permittivity of the prism, metal, and dielectric. *ω* and *θ_in_* are the light frequency and incident angle.

Along the *z*-axis, the different layers of the SPR device are stacked. The relative permittivity and thickness of each layer are *ε*_i_ and *d*_i_. In a mathematical form, the fields at the first interface (z = 0) can be represented in terms of those at the final interface (z = z_N–1_) as

(6) $$\left[\begin{array}{c} E_{1}\\ H_{1} \end{array}\right]=D_{T}{\left[\begin{array}{c} E_{N-1}\\ H_{N-1} \end{array}\right]}_{\;}$$

where *E*_1_ and *E_N_*_-1_ are the tangential electric fields at the first and the last boundaries. *H*_1_ and *H*_N-1_ are the same for the magnetic field. *D_T_* is the transfer matrix. The characteristic matrix (*D_j_*) of the jth layer is given as

(7) $$D_{j}=\left[\begin{array}{cc} \cos\left(Y_{j}\right) & -\frac{i\;\sin\left(Y_{j}\right)}{U_{j}}\\ -iU_{j}\sin\left(Y_{j}\right) & \cos\left(Y_{j}\right) \end{array}\right]$$

$Y_{j}$ is the phase shift which is given by

(8) $$Y_{j}=\frac{2\pi }{\lambda }d_{j}{\left(\varepsilon_{j}-{\left(n_{1}\sin\theta_{1}\right)}^{2}\right)}^{1/2}$$

where *n*_1_ and *θ*_1_ are the prism RI and incident angle.

For TM waves, *D_j_* is defined as

(9) $$U_{j}={\left(\varepsilon_{j}-{\left(n_{1}\sin\theta_{1}\right)}^{2}\right)}^{0.5}/\varepsilon_{j}$$

The system TM (*D_T_*) of the whole structure can be expressed as the product of the individual matrices

(10) $$D_{T}=D_{Ag}\;D_{S}\;D_{M}=\left[\begin{array}{cc} D_{11} & D_{12}\\ D_{21} & D_{22} \end{array}\right]$$

where $D_{Ag}$, $D_{S}$, and $\;D_{M}$ are the TMs of the Ag, Si, and Ti_3_C_2_T_x_ layers.

In terms of the system transfer matrix elements *D_ij_*, the reflection coefficient (*r*) is given by

(11) $$r=\frac{\left(D_{11}+D_{12}U_{N}\right)U_{1}-\left(D_{21}+D_{22}U_{N}\right)}{\left(D_{11}+D_{12}U_{N}\right)U_{1}+\left(D_{21}+D_{22}U_{N}\right)}$$

The reflectance (*R*) of the proposed SPR nanostructure

(12) $$R=r.r^{*}={\left|r\right|}^{2}$$

Sensitivity (*S*), detection accuracy (DA), full width at half maximum (FWHM), and figure of merit (FoM) are commonly calculated to estimate the performance of sensors. The modification in the RI of the analyte medium (Δ*n*) leads to a shift in the resonance angle (Δθ*_res_*). In an SPR sensor, the sensitivity is usually determined by both Δ*n* and Δ*θ_re_*_s_ in a unit of degree/RIU as [25,26,36]

(13) $$S=\frac{\Delta \theta_{res}}{\Delta n}\;\;\;\;\;\;$$

In photonic crystal-based sensors, the sensitivity is calculated in the unit of nm/RIU, but in SPR-based sensors, the sensitivity is calculated in the unit of degree/RIU since the resonance dip changes its angular position for any change in the analyte RI.

The reflectance curve can also be used to calculate the *FWHM*. It can be calculated using the formula

(14) $$FWHM=\theta_{2}-\theta_{1}\;\;\;\;$$

where *θ_1_* and *θ*_2_ are the resonance angles at 50% reflectance.

DA can be obtained as [37]

(15) $$DA=\frac{1}{FWHM}$$

The *FoM* is the product of *S* and *DA* of the sensor [37]:

(16) FoM=S×DA    

## 3. Results and Discussion

An SPR structure-based optical sensor was investigated for sensing fat concentration in milk. All the following calculations are conducted with λ = 632.8 nm. The RIs are determined using the formulae described in Section 2 at λ = 632.8 nm. It is found that $n_{p}=1.5151$, n_Ag_ = 0.05621+4.2777 i, n_S_ = 3.8468, and n_M_ = 2.38+1.33 i for BK7, Ag, Si, and Ti_3_C_2_T_x_ materials. The RI of a material deposited in a vacuum chamber depends on many evaporation parameters, such as pressure, temperature, evaporation method, evaporation rate, and the thickness of the evaporated layer. Usually, when theoretical SPR structures are presented, these parameters are not considered and only the wavelength dependence is taken into account [7,18]. The layer thicknesses were initially chosen as d_Ag_ = 50 nm, d_S_ = 1 nm, and d_M_ = L × 0.993 nm with L being the number of Ti_3_C_2_T_x_ layers (we initially used L = 1). In Ref. [38], the RIs of milk samples at various fat concentrations are provided. These values are based on the experimental results and when plotted, we found that the relation between RI and concentration is not linear except for high values of concentration.

**Figure 2:**
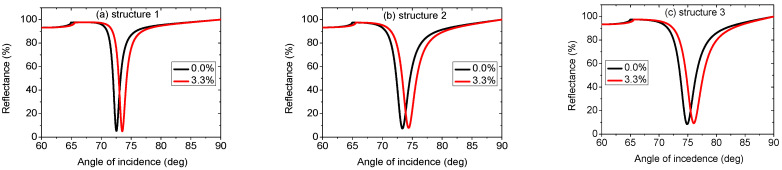
Reflectance curves of (**a**) prism/Ag/analyte medium, (**b**) prism/Ag/Ti_3_C_2_T_x_/analyte medium, and (**c**) prism/Ag/Si/Ti_3_C_2_T_x_/analyte medium.

In Figure 2, the reflectance curves for three sensor architectures are shown for two different fat concentrations in a milk sample. The results of the three structures show that the resonance dip shifts towards higher resonance angles as the fat concentration in milk increases. Figure 2a plots the reflectance curve of the traditional SPR sensor with the structure prism/Ag/analyte medium (structure 1). A resonance angle of 72.56 deg. is found for a sensing medium of RI of 1.3452. A tiny increment in the RI of the analyte medium (Δn = 0.0065) leads to a resonance angle of 73.53 deg. Using Equation (13), the sensitivity is found for the conventional SPR sensor as 149.23 deg./RIU. FWHM, DA, and FoM are obtained from the resonance curve (Figure 2a) as 1.35 deg., 0.740 deg.^−1^, and 110.43 RIU^−1^, respectively. After inserting a monolayer of Ti_3_C_2_T_x_ in the traditional SPR sensor, the structure becomes prism/Ag/Ti_3_C_2_T_x_/analyte medium (structure 2). Figure 2b depicts the structure’s reflectance curve. The sensitivity achieved is 152.77 deg./RIU, FWHM, and DA, and the FoM achieved for structure 2 are 2.3 deg., 0.434 deg.^−1^ and 66.30 RIU^−1^, respectively. The sensitivity improvement as a result of inserting a monolayer of Ti_3_C_2_T_x_ between the analyte and metal layers is 2.37%. This can be attributed to excellent an absorption property and chemical stability. Moreover, as a result of its larger surface area, Ti_3_C_2_T_x_ has a greater potential to bind biomolecules [39]. Introducing a 1 nm layer of Si material between the 50 nm layer of Ag and the monolayer of Ti_3_C_2_T_x_, the structure becomes prism/Ag/Si/Ti_3_C_2_T_x_/analyte medium (structure 3) and the resonance curve takes the form shown in Figure 2c. An enhanced sensitivity of 180 deg./RIU is attained with a sensitivity improvement of 20.61% and 17.82% over the conventional SPR (without Ti_3_C_2_T_x_ and Si) and the proposed SPR without Si (including Ti_3_C_2_T_x_), respectively. The sensitivity improvement of structure 3 is attributed to the use of high RI material at the analyte medium interface. Inserting a silicon layer leads to the field intensity enhancement at the analyte medium interface. To detect a material efficiently, the field intensity at the material interface should be as high as possible. FWHM, DA, and FoM are obtained from the resonance curve (Figure 2c) as 2.84 deg., 0.352 deg.^−1^, and 63.36 RIU^−1^, respectively. Performances for all structures are summarized in Table 2.

**Figure 3:**
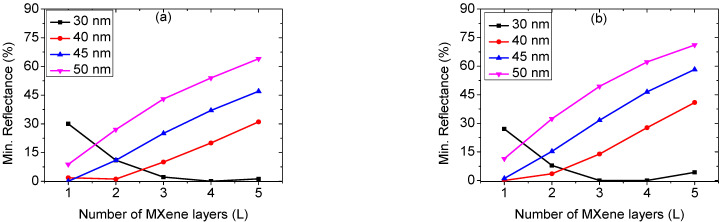

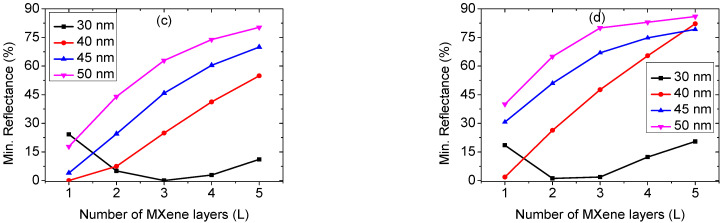
Minimum reflectance variation of the structure with the number of Ti_3_C_2_T_x_ sheets for various thicknesses of Ag at Si thickness of (**a**) 1, (**b**) 2, (**c**) 3, and (**d**) 4 nm.

We examined different combinations of metal and Si thicknesses to develop the performance of the proposed sensor. We also examined different numbers of Ti_3_C_2_T_x_ layers. Minimum (Min.) reflectance and sensitivity are calculated from the resonance curves for each of these combinations. The metal layer thickness is varied in steps of 5 nm from 30 nm to 50 nm, whereas the Si thickness is changed in steps of 1 nm from 1 nm to 4 nm. The number of Ti_3_C_2_T_x_ is varied between 1, 2, 3, 4, and 5 layers. Figure 3 shows how the minimum reflectance varies with the number of Ti_3_C_2_T_x_ sheets for various thicknesses of the Ag layer. The different panels of Figure 3 are plotted at different Si thicknesses of 1 nm (Figure 3a), 2 nm (Figure 3b), 3 nm (Figure 3c), and 4 nm (Figure 3d). From Figure 3a–d, at a Ag thickness of 30 nm, minimum reflectance first decreases near zero and then begins enhancing for a greater number of Ti_3_C_2_T_x_ layers. For other thicknesses of Ag, it is seen that reflectance curves show a continuous increase with the increasing number of Ti_3_C_2_T_x_ layers. From analyzing Figure 3a–d, at a silver layer thickness of 30 nm, we can conclude that the minimum reflectance tends to zero out at a lower number of Ti_3_C_2_T_x_ sheets as the thickness of the Si layer increases. For higher values of the silver layer thickness, the minimum reflectance increases with the increasing Si layer thickness. This suggests that for complete energy transfer to surface plasmons at the Ag interface, any decrease in Si or Ag layer thickness is reimbursed by an increase in the number of Ti_3_C_2_T_x_ sheets [40]. As a result, both the Si and Ti_3_C_2_T_x_ layers are acting as absorption media in this case, allowing energy to be transferred completely to the silver film. The reflectance curves corresponding to a Ag layer thickness of 30 nm differ from those corresponding to thicknesses of 40, 45, and 50 nm. This means that the reflectance dependence on metal thickness is essential and crucial. We tried a metal layer thickness of 20 nm, and no dip was observed in the reflectance curve.

**Figure 4:**
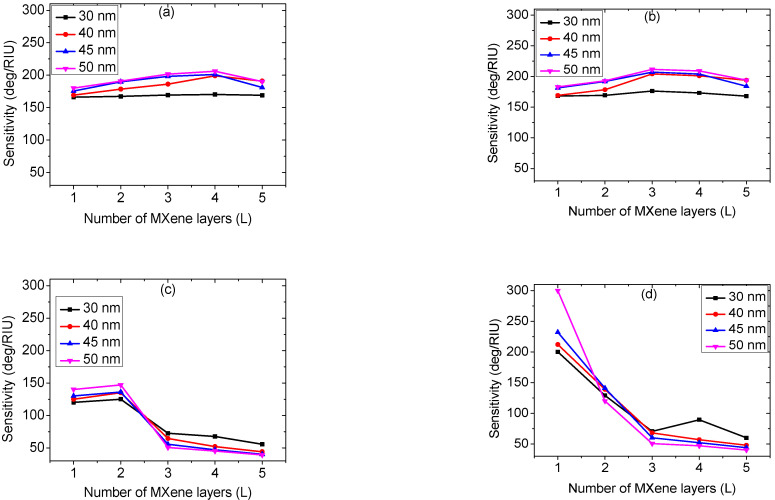
Sensitivity versus the number of Ti_3_C_2_T_x_ sheets with various thicknesses of Ag layer at Si thicknesses of (**a**) 1, (**b**) 2, (**c**) 3, and (**d**) 4 nm.

Figure 4 depicts the refractometric sensor sensitivity as a function of the number of Ti_3_C_2_T_x_ sheets for various metal layer thicknesses. The number of Ti_3_C_2_T_x_ sheets varies between 1, 2, 3, 4, and 5 layers, whereas the metal layer thickness is taken from 30 nm to 50 nm in 5 nm steps. The different panels of Figure 4 are plotted at different Si layer thicknesses of 1 nm (Figure 4a), 2 nm (Figure 4b), 3 nm (Figure 4c), and 4 nm (Figure 4d). As is evident, the sensitivity enhances with the increase in the number of Ti_3_C_2_T_x_ sheets, reaches the highest value, and then decays after a further increase of Ti_3_C_2_T_x_ sheets. Figure 4a–d shows that the sensitivity peak shifts to a lower number of Ti_3_C_2_T_x_ sheets as the Si layer thickness increases. These results were obtained because of the substantial transfer of the charge from the interface separating Ti_3_C_2_T_x_ and Si from the metal film for a higher number of Ti_3_C_2_T_x_ layers. As can be seen from Figure 4d, the highest sensitivity of 300.01 deg./RIU is noticed at 4 nm Si layer thickness. As a result, the suggested SPR refractometric sensor with Ag (50 nm), Si (4 nm), and Ti_3_C_2_T_x_ (monolayer) is the optimal choice for a ultra-high sensitivity sensor.

**Figure 5:**
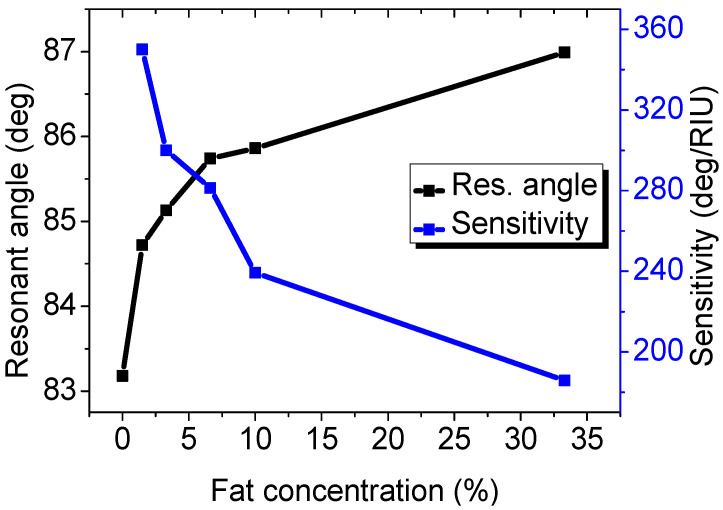
Resonance angle and sensitivity versus the fat concentration.

Small changes in the RI of the analyte lead to a significant shift in the angular position of the reflectance dip. We analyzed the resonance angle shift and sensitivity of the proposed sensor (Ag of 50 nm/Si of 4 nm/a monolayer of Ti_3_C_2_T_x_) for varied fat concentrations (0.0%, 1.5%, 3.3%, 6.6%, 10%, and 33.3%) in milk. The fat concentrations in milk and the corresponding RI, angular position of the resonance dip, shift of the resonance angle, and sensitivity are summarized in Table 3. Figure 5 shows that any small change in the fat concentration, and hence in the RI, can be easily sensed, which reflects a shift in the resonance dip angular position. The figure also shows that increasing the fat concentration leads to a drop in the sensitivity of the sensor. The sensitivity is found to be 350, 300.01, 281.32, 239.29, and 185.85 deg./RIU for fat concentrations of 0.0%, 1.5%, 3.3%, 6.6%, 10%, and 33.3%, respectively. The maximum sensitivity of 350 deg./RIU can be found at a fat concentration of 1.5% in milk. The sensitivity drop with the increase in the milk fat concentration can be attributed to an increase in the index of refraction of the analyte. Therefore, the proposed sensor can be employed efficiently for detecting the fat concentration in milk.

It is worth mentioning that raising the milk temperature leads to a lower RI. Since milk gets less dense and viscous at higher temperatures, light travels faster through the milk. We can compare this to Figure 5. In Figure 5 and Table 3, as the fat concentration increases, the RI of the milk increases and the sensitivity decreases. This leads to the following conclusion: as the RI gets lower due to the temperature increase, the sensitivity gets higher.

**Figure 6:**
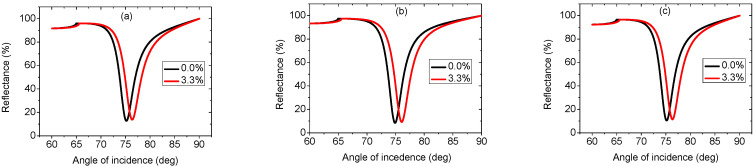
Reflectance curves of the structure prism/Ag/Si/Ti_3_C_2_T_x_/analyte medium for different values of the damping rate. (**a**) γ = 11.7809 × 10^−13^ rad/s, (**b**) γ = 10.7040 × 10^−13^ rad/s, and (**c**) γ = 9.8072 × 10^−13^ rad/s.

The refractive index of the metal layer can be written in terms of the wave frequency (ω) as $n_{Ag}^{2}=1-\frac{\omega_{p}{}^{2}}{\omega \left(\omega +i\gamma \;\right)}$, where $\omega_{p}$ is the plasma frequency and γ is the damping rate. It is worth investigating the effect of the damping rate on the sensitivity. In Figure 6, the reflectance curves of the structure prism/Ag/Si/Ti_3_C_2_T_x_/analyte medium are shown for different values of the damping rate. We considered the following values of the damping rate: 11.7809 × 10^−13^, 10.7040 × 10^−13^_,_ and 9.8072 × 10^−13^ rad/s. The sensitivity was found to be 181.54, 180, and 178.43 deg./RIU corresponding to γ = 11.7809 × 10^−13^, γ = 10.7040 × 10^−13^_,_ and γ = 9.8072 × 10^−13^ rad/s, respectively. It is clear that increasing the metal damping rate can improve sensitivity, and this is in agreement with Ref. [41].

To compare the results of the present work with recent SPR refractometric sensors utilizing Ti_3_C_2_T_x_ and other 2D nanomaterials, Table 4 is presented, which shows that the sensitivity of the model used in the current work is the highest.

## 4. Conclusions

An SPR-based refractometric sensor was theoretically proposed for sensing fat concentration in milk. The proposed configuration employs BK7 prism, silver, Si, and Ti_3_C_2_T_x_ materials. Ti_3_C_2_T_x_ 2D nanomaterial was proved to facilitate the interaction of biomolecules and enhance adsorption in SPR structures due to its exceptional optical and electrical properties. Si, as a high-index material, can improve the sensitivity of a refractometric sensor by enhancing TM wave intensity at the sensing interface. We first examined the sensor performance for the structures prism/Ag/analyte medium (structure 1), prism/Ag/Ti_3_C_2_T_x_/analyte medium (structure 2), and prism/Ag/Si/Ti_3_C_2_T_x_/analyte medium (structure 3), and found that structure 3 had the utmost sensitivity of 180 deg./RIU with a sensitivity improvement of 20.61% and 17.82% over structure 1 and structure 2, respectively. The reflectivity and sensitivity of the third structure were investigated with a number of Ti_3_C_2_T_x_ sheets. It was also studied with different thicknesses of Si and silver layers. We found that when the Ag layer thickness was kept constant, the sensitivity peak shifted toward a lower number of Ti_3_C_2_T_x_ sheets as the thickness of the Si layer grew. We also found that the angular sensitivity could be enhanced when the thickness of the Ag layer grew. Sensitivity was also investigated with variations in the fat concentration of milk. Different fat concentrations of 0.0%, 1.5%, 3.3%, 6.6%, 10%, and 33.3% were considered, and the highest sensitivity was found at a concentration of 1.5%. The parameters at which sensitivity is maximum (350 deg./RIU) are as follows: silver (50 nm), Si (4 nm), a monolayer of Ti_3_C_2_T_x_, and fat concentration of 1.5%. The proposed structure can be applied efficiently for the detection of low-index chemicals and biomolecules by simply adding the analyte as a sensing medium to the structure shown in Figure 1 [46,47,48].

It is of great significance to provide some advice regarding the fabrication of the proposed sensor. A thin film of Ag (50 nm) is deposited on the top of BK7 prism. The following deposition methods are usually employed: vacuum thermal evaporation, electron beam evaporation, ion plating evaporation, and laser beam evaporation, etc. A 4 nm thin film of silicon is grown on the Ag film. The following deposition methods can be used for a Si film: photo CVD, thermal CVD, HOMO (hot reactor and cold substrate) CVD, and hot-wire CVD. A monolayer of Ti_3_C_2_T_x_ is then deposited on the Si film using CVD or electrospinning techniques.

## Figures and Tables

**Table 1:** The coefficients *d_i_* and *e_i_* of the BK7 prism.

The Coefficient *d_i_*	Value of *d_i_*	The Coefficient *e_i_*	Value of *e_i_*
*d*_1_	1.03961212	*e*_1_	0.00600069867
*d*_2_	1.01046945	*e*_2_	103.560653
*d*_3_	0.231792344	*e*_3_	0.0200179144

**Table 2:** The used structures and their performance parameters at d_Ag_ = 50 nm, d_S_ = 1 nm, and d_M_ = L × 0.993 nm where L = 1.

Structure No.	The Used Structure	FWHM (deg.)	DA (deg.^−1^)	S (deg./RIU)	FOM (RIU^−1^)
1	Ag	1.35	0.740	149.23	110.43
2	Ag/Ti_3_C_2_T_x_	2.3	0.434	152.77	66.30
3	Ag/Si/Ti_3_C_2_T_x_	2.84	0.352	180	63.36

**Table 3:** Fat concentration with the corresponding RI, shift in resonance angle, and sensitivity.

Fat Concentration (%)	RI	Resonance Angle (deg.)	Shift in Resonance Angle (deg.)	Sensitivity (deg./RIU)
0	1.3452	83.18	-	-
1.5	1.3496	84.72	1.54	350
3.3	1.3517	85.13	1.95	300.01
6.6	1.3543	85.74	2.56	281.32
10	1.3564	85.86	2.68	239.29
33.3	1.3657	86.99	2.77	185.85

**Table 4:** A comparison between the current work and most recent published works.

Structure	Year	Sensitivity (deg./RIU)	Reference
An SPR biosensor with ZnO, Ag, and G layers.	2018	187.43	[42]
An SPR biosensor using 3 sheets of G.	2019	121.67	[43]
An SPR biosensor employing Ti_3_C_2_T_x_ and BP.	2019	190.22	[26]
An SPR biosensor employing a thin layer of ZnO for DNA hybridization.	2020	156.33	[44]
An SPR sensor employing a thin layer of plasma.	2021	103	[45]
An SPR biosensor based on G.	2022	199.87	[32]
Detection of fat concentration in milk using SPR biosensor based on Si and Ti_3_C_2_T_x_.	2022	350	Current work

## Data Availability

Data will be made available on reasonable request.
